# Molecular-level investigation of plasticization of polyethylene terephthalate (PET) in supercritical carbon dioxide via molecular dynamics simulation

**DOI:** 10.1098/rsos.220606

**Published:** 2022-08-24

**Authors:** Fayu Sun, Hu Dedong, Li Fei, Wang Weiqiang, Gao Zhaotao, Zhang Zhuo

**Affiliations:** ^1^ Key Laboratory of High-efficiency and Clean Mechanical Manufacture (Ministry of Education), National Experimental Teaching Demonstration Center for Mechanical Engineering, School of Mechanical Engineering, Shandong University, Jinan 250061, People's Republic of China; ^2^ Shanda-Lunan Research Institute of Supercritical Fluid Technology, Shandong University, Jinan 250061, People's Republic of China; ^3^ School of Electromechanical Engineering, Qingdao University of Science and Technology, Qingdao 266061, People's Republic of China

**Keywords:** molecular simulation, polyethylene terephthalate, plasticization, glass transition temperature, supercritical carbon dioxide

## Abstract

The current study aims to use the molecular dynamics (MD) simulation method to discuss the glass transition behaviour and fractional free volume (*FFV*) of the pure polyethylene terephthalate (PET) and the plasticized PET induced by supercritical carbon dioxide (SC-CO_2_) sorption. The adsorption concentration reproduced through sorption relaxation cycles (SRC) was firstly estimated and in an order of magnitude with the known experimental results available in the reported literature. The *FFV* induced by SC-CO_2_ in PET polymer changes regularly, which is proportional to the capacity of SC-CO_2_ adsorption with the changes in temperature and pressure. The glass transition temperature (*T*_g_) was further estimated to be almost identical to the known experimental values and shows a gradually decreasing tendency with the increase of pressure. Meanwhile, the plasticization of PET polymer studied by radial distribution functions showed that CO_2_ molecules occupying the sorption sites on the PET backbone promoted plasticization by increasing the fluidity of the PET backbone chain.

## 1. Introduction

In recent years, supercritical carbon dioxide (SC-CO_2_) as a solvent existing above its critical point (*T*_c_ = 31°C, *Pc* = 7.38 MPa) has attracted considerable attention in many fields [1], such as pharmaceutical, blending [2], impregnation [3], foaming [4], extracting [5] and dyeing [6]. SC-CO_2_ is a highly compressed fluid with numerous unique characteristics including high diffusion coefficient, zero surface tension and low viscosity. Meanwhile, SC-CO_2_ has become the most widely used solvent as it's low cost, recyclable, non-toxic, chemically inert and non-flammable [7]. Many polymer-related processes have been developed by the fact that the sorption of CO_2_ by polymer leads to reducing the glass transition temperature of polymers and the swelling and plasticizing effects, resulting in the changes in the mechanical and physical properties of the polymer [6], such as material modification, the grade of polymer, small molecule penetration, polymer flick, supercritical fluid dyeing and superfine material preparation, etc [8].

A great number of studies have been published on the swelling and mechanical and physical properties of PET in SC-CO_2_. Chen *et al*. [9] investigated the solubility of CO_2_ in solid PET and showed a linear relationship with Henry's law at low pressure (less than 8 MPa), and the solubility decreased with the increase of temperature at high pressure. Chen [10] compared the effects of temperature (50 ∼ 120°C) and pressure (0.1 ∼ 20 MPa) on the swelling rate of PET and obtained the polymer glass transition temperature in the supercritical fluid environment by their self-made high pressure autoclave with a view window, which detected the length change of PET film in the CO_2_ state via a CCD camera. Han *et al*. [6] measured the swelling of polyester yarns under different conditions and found that the maximum theoretical value was 0.725 mm under the optimum treatment conditions (*T* = 140°C, *p* = 26 MPa, *t* = 60 min) using the response surface methodology. Hirogaki [11] investigated the influence of SC-CO_2_ and the effects of additive modifiers on the swelling behaviour of PET, and found that SC-CO_2_ promoted the crystallization of PET polymer, but the additional modifiers (such as alcohols) prevented the crystallization of SC-CO_2_.

In recent years, the application of SC-CO_2_ dyeing has attracted wide industrial and academic attention. It mainly includes the solubility of dyes [12], dyeing process, dye-design [13] and dyeing equipment development [14]. At present, the dyeing of polyethylene terephthalate fibre (PET) in SC-CO_2_ has been industrialized [15]. In the process of SC-CO_2_ dyeing, due to the dissolution of CO_2_ in polymer, the swelling and mechanical and physical properties of PET improved the dyeing quality of polymer, since the swelling behaviour of polymer is directly related to its dyeability [6]. The molecular simulation has become an alternative approach to examining the glass transition temperature (*T*_g_) [16], but no studies have as yet investigated the plasticization in PET polymer by simulation.

In this research, molecular dynamics (MD) simulation was used to investigate the effects of CO_2_ on the *T*_g_ and fractional free volume (FFV) of polyethylene terephthalate (PET) at different temperatures and pressures.

## 2. Computational details

### 2.1. Model building

**Figure 1.**
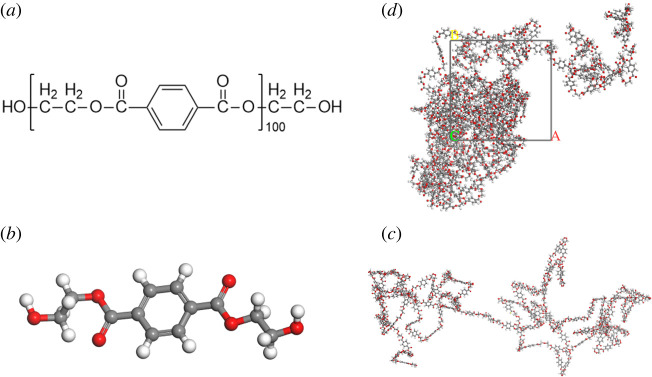
Illustration of calculation model of PET: (*a*) chemical structure (*b*) molecular monomers (*c*) molecular chain (100) (*d*) cell model.

In this work, all the molecular simulation processes were completed by Accelrys Material Studio (MS) 2017 [17]. The PET chain consisting of 100 repeat units [18] and CO_2_ molecules were built by materials visualizer modules [19]. The cubic cells with two PET chains were constructed using an amorphous cell module, which is shown in figure 1, and were subsequently equilibrated using the Forcite module. In order to avoid chain rings catenation and spearing, the initial densities of cubic cells were set at 0.3 g cm^−3^, 512 methanol molecules were added randomly into the cells [20], and the methanol molecules were deleted before the cells were started. The COMPASS (condensed-phase optimized molecular potentials for atomistic simulation studies) force field [21] was used for all simulations. Andersen thermostats and Berendsen barostats were used to control the temperature and pressure respectively in all simulations. The non-bonded van der Waals interactions were calculated by the Atom based method with a cutoff distance of 18.5 Å, a spline width of 1 Å, and a buffer width of 0.5 Å. The non-bonded electrostatic interactions were characterized via the Ewald method with accuracy of 10^−5^ kcal mol^−1^.

### 2.2. Simulation method

**Figure 2.**
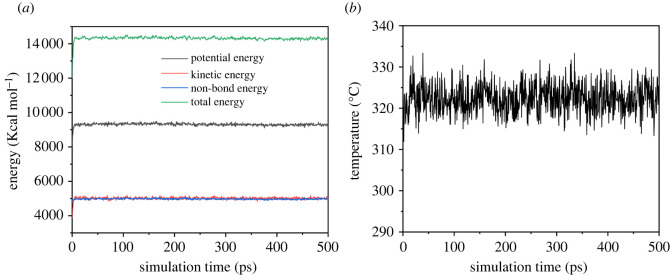
Energy and temperature fluctuations observed during molecular dynamic simulation of 500 ps for PET (*a*) energy fluctuations (*b*) temperature fluctuations.

All the initial polymer chains, CO_2_ molecular and cubic cells were subjected to energy minimization to remove the local non-equilibrium by geometry optimization with 10 000 steps using the Smart algorithm [17]. Firstly, the systems were subjected to MD runs in *NPT* ensemble with 1fs time step for a total simulation time of 500 ps using the Forcite module [17] to equilibrate and remove residual stress. Secondly, the systems were subjected to 500 ps *NVT* dynamics and 1000 ps *NPT* dynamics at room temperature and pressure. Finally, the systems were subjected to 300 ps *NPT*, 300 ps *NVT* and 500 ps *NPT* dynamics calculation at different temperatures. Generally, the system reaches equilibrium when the standard deviation of the energy and temperature requirements of the system is less than 10%. As presented in figure 2, the system reached equilibrium after the NPT dynamic simulation of 500 ps.

The sorption module of Materials Studio 2017 was applied to study the plasticated PET induced by CO_2_ sorption. The simulation was evaluated by the Grand Canonical Monte Carlo (GCMC) ensemble at ‘fixed pressure’ task, which fixed temperature, volume and chemical potential using the Metropolis Algorithm [22]. For each case, it provides three types of operations with equal probability in the cell: displacement, creation and deletion. The total potential energy E (before one operation), E’ (after the certain operation) and the variation ΔE = E′–E are calculated from equation (2.1). Next, according to the assumed acceptance probabilities, this operation is accepted or rejected. The acceptance probabilities are min $\left\{1,\;\exp[-\Delta E/k_{B}T]\right\}$ and min $\left\{1,\;(\lambda^{3}N_{i}/V_{i})\exp[-(\mu +\Delta E)/(k_{B}T)]\right\}$, respectively, where $\lambda =\sqrt{h^{2}/(2\pi mk_{B}T)}$, *h* is the Planck constant, *m* is the mass of CO_2_, *T* is the temperature, *µ* is the chemical potential, *V_i_* is the volume of the ith subspace and N_i_ is the number of CO_2_ molecules in the ith subspace.

The temperature and pressure points were selected according to the experimental data values in the related literature [10]. For the sake of reproducing volume swelling and plasticization induced by sorption, sorption–relaxation cycles (SRC) [16,23] were used until the sorption coefficients converge. The relaxation process: at each cycle, the cell was loaded with CO_2_ molecules at the considered temperature and pressure by GCMC ensemble, and then the cell was relaxed by running a 300 ps NPT molecular dynamic equilibration. Next, the cell containing CO_2_ molecules was loaded with CO_2_ molecules until the sorption coefficients converge.

2.1 $$\begin{array}{ll} & E=\sum_{b}K_{2}{\left(b-b_{0}\right)}^{2}+K_{3}{\left(b-b_{0}\right)}^{3}+K_{4}{\left(b-b_{0}\right)}^{4}\\ & \;+\sum_{\theta }{H_{2}(\theta -\theta_{0})}^{2}+H_{3}{\left(\theta -\theta \right)}^{3}+H_{3}{\left(\theta -\theta \right)}^{3}\\ & \;+\sum_{\phi }\left\{V_{1}[1-\cos(\phi -\phi_{1}^{0})]+V_{2}[1-\cos(2\phi -\phi_{2}^{0})]+V_{3}[1-\cos(3\phi -\phi_{3}^{0})]\right\}\\ & \;+\sum_{\chi }K_{\chi }\chi^{2}+\sum_{b}\sum_{b^{\prime }}F_{bb^{\prime }}(b-b{}_{0})(b^{\prime }-b_{0}^{\prime })+\sum_{\theta }\sum_{\theta^{\prime }}F_{\theta \theta^{\prime }}(\theta -\theta_{0})(\theta^{\prime }-\theta_{0}^{\prime })+\sum_{b}\sum_{\theta }F_{b\theta }(b-b_{0})(\theta -\theta_{0})\\ & \;+\sum_{b}\sum_{\phi }(b-b_{0})(V_{1}\cos\phi +V_{2}\cos2\phi +V_{3}\cos3\phi )+\sum_{b^{\prime }}\sum_{\phi }\left(b^{\prime }-b_{0}^{\prime })(V_{1}\cos\phi +V_{2}\cos2\phi +V_{3}\cos3\phi \right)\\ & \;+\sum_{\theta }\sum_{\phi }\left(\theta -\theta_{0}\right)(V_{1}\cos\phi +V_{2}\cos2\phi +V_{3}\cos3\phi )+\sum_{\phi }\sum_{\theta }\sum_{\theta^{\prime }}K_{\phi \theta \theta^{\prime }}\cos\phi (\theta -\theta_{0})(\theta^{\prime }-\theta_{0}^{\prime })\\ & \;+\sum_{i>j}\frac{q_{i}q_{j}}{\varepsilon r_{ij}}+\sum_{i>j}(\frac{A_{ij}}{r_{ij}^{9}}-\frac{B_{ij}}{r_{ij}^{6}}). \end{array}$$

## 3. Results and discussion

### 3.1. Sorption of CO_2_ in PET

**Figure 3.**
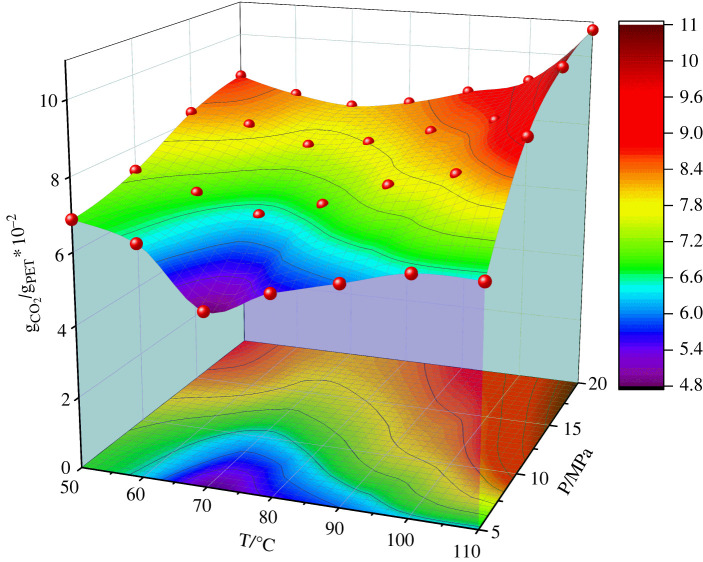
Adsorption concentration for CO_2_ in PET.

In this study, the sorption equilibrium of CO_2_ in PET at different pressures and temperatures has been simulated using SRC as shown in §2.2. From figure 3 we can see that the adsorption concentration of PET was in the ranges of pressure from 5 to 20 MPa and temperature from 50 to 110°C. The simulated adsorption concentration of CO_2_ in PET was generally in an order of magnitude with the known experimental results available in the reported literatures as show in table 1. The simulation results were approximately three times larger than the experimental results [24]. In the simulating process, the authors only established the amorphous region, but did not establish the crystallization region, which resulted in more CO_2_ molecules absorbed into the amorphous region compared with the real PET fibre. The adsorption concentration of CO_2_ in PET decreases first and then increases gradually with the increase of temperature. Meanwhile, the decrease of the slope is gradually smaller and the increase of the slope is steeper and steeper as the pressure goes up. The tendency seems to follow the literature results [24]: at the lower densities, the adsorption concentration decreases with the increase of temperature. However, as the pressure increases, the inflection point of the change in the tendency is going up earlier and earlier. When the pressure is constant, the increase of temperature results in the decrease of CO_2_ density, which decreases the number of molecules of CO_2_ in the amorphous region (free volume). However, when the temperature is close to the point of *T*_g_, the PET molecular chain amplitude increases, and the amorphous region becomes larger, resulting in more CO_2_ molecules entering the amorphous region. The adsorption concentration of CO_2_ in PET near of the point of *T*_g_ starts to increase. When the temperature is constant, the adsorption concentration of CO_2_ increases with the increase of pressure. The CO_2_ density increases with the increase of pressure, causing more CO_2_ molecules to enter the amorphous region of PET fibre. The PET molecular chain was intensified due to the intensified movement of CO_2_ molecules, leading to the enlargement of amorphous region and more CO_2_ molecules being absorbed into PET. As the pressure increases, the movement of CO_2_ molecules becomes gradually stronger, causing the intensified movement of the PET molecular chain. The PET molecular chain begin to unfreeze at a lower temperature, leading to the increase of free volume and promoting the adsorption of more CO_2_ molecules. As shown in figure 3, the inflection point of the change in the tendency is shifted to the left, and consistent with the decrease of glass transition temperature with the increase of pressure (§4.4 will discuss the relationship between glass transition temperature and pressure).

**Table 1.** The simulated and experimental adsorption concentration (g_co2_/g_PET_) for CO_2_ in PET.

P/MPa T/°C	5		10		15		20	
	Sim	Exp	Sim	exp	Sim	exp	Sim	exp
50	6.874	*	7.332	*	8.249	*	8.707	*
60	6.416	*	6.874	*	8.02	*	8.249	*
70	4.812	*	6.416	*	7.561	*	8.02	*
80	5.499	*	6.874	2.2	7.790	*	8.249	*
90	5.957	*	7.561	*	8.249	*	8.707	*
100	6.416	1.25	8.02	2.1	8.707	*	9.165	2.6
110	6.416	*	9.165	*	10.311	*	10.769	*

### 3.2. Swelling of the PET polymer

The swelling and plasticization of the PET polymer is a long process and it is a difficult task to figure out the specific mechanism through experiments under high pressure. However, molecular simulation has provided a unique opportunity to examine the swelling behaviour and plasticization of the PET polymer in the SC-CO_2_ atmosphere. The sorption of CO_2_ into PET polymer induced volume swelling and plasticization, causing the decrease of glass transition temperature. It has been shown that the reason for plasticization is the interactions between CO_2_ and the polymer, and plasticization disrupts chain packing and enhances the inter-segmental mobility of polymer [25].

**Figure 4.**
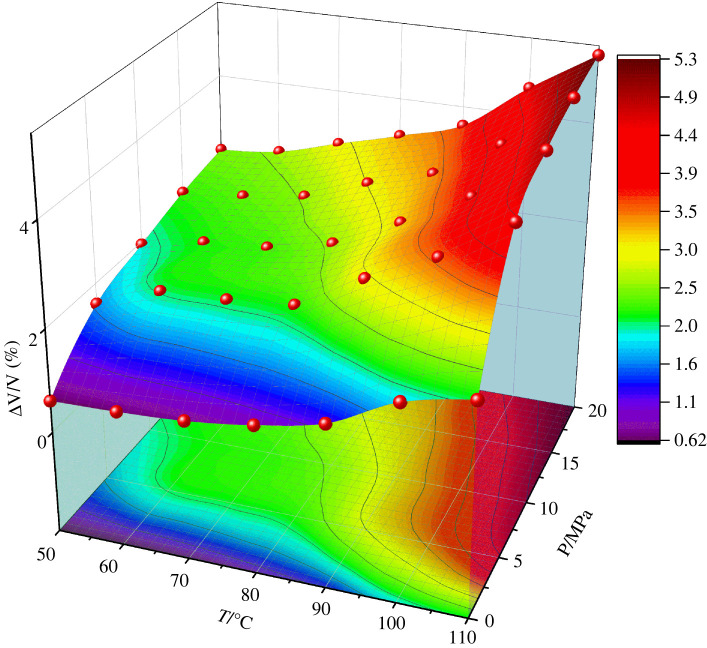
The curves of volume change with temperature for the PET at different pressures.

Figure 4 shows the volume change of PET polymer at the pressures ranging from 5 to 20 MPa and the temperatures ranging from 50 to 110 °C. It can be seen that the volume change increases and the slope of the volume change increases gradually with the increase of temperature in the meantime. Meanwhile, with the increase of pressure, the increase of the slope becomes larger and larger. For example, the volume change increases from 0.62% to 2.1% at atmospheric pressure and the temperature ranging from 50 to 110°C. Meanwhile, the volume change increases from 0.62% to 2.25% at the temperature of 50°C and the pressure rising from 0 to 15 MPa. The swelling of the above two is basically the same. Thus it is feasible to use SC-CO_2_ for swelling and other related processes. In the process of development, the same effect is achieved by adjusting the range of pressure change at a lower temperature, which can save energy consumption and protect the environment.

### 3.3. Fractional free volume

The free volume theory was first proposed by Fox and Flory in 1950 [26]. According to the theory, the volume of liquid or solid consists of two parts: one is the volume occupied by molecules and the other is the unoccupied free volume. The size, morphology and spatial size distribution of free volume play an important role in the diffusion behaviour of molecules in polymer, providing the necessary space for diffusion molecules. In general, the larger the *FFV* of the polymer, the larger the diffusion coefficient of diffusing material will be. The free volume of the system is obtained by the hard ball probe method, which is using the ‘Atom Volume & Surface’ tool in MS software package. When the hard ball probe with a radius of *R_P_* = 0 moves on the van der Waals surface of PET polymer, the volume surrounded by the Connolly surface formed by the contact point is the free volume. The *FFV* of the system can be obtained by dividing the simulated free volume by the total volume. The relationship between *FFV* and temperature of PET polymer at different pressures is shown in figure 4.

**Figure 5.**
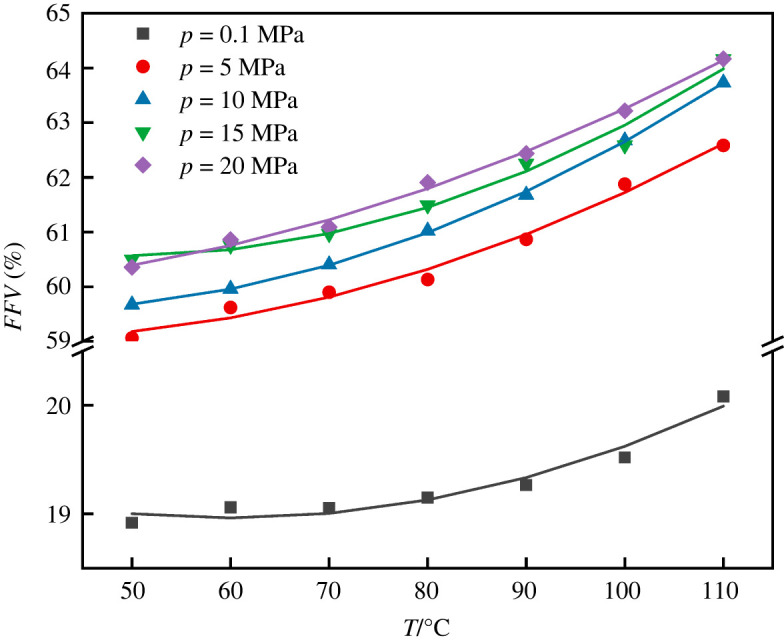
The relationship between free volume fraction (*FFV*) and temperature of PET at different pressures.

From figure 4 we can see the *FFV* for PET change at the pressures ranging from 0.1 to 20 MPa and the temperatures ranging from 50 to 110°C. It can be shown that the *FFV* increases and the slope of the *FFV* starts to increase gradually near the *T*_g_ with the increase of temperature, corresponding to adsorption concentration for CO_2_ in PET. The *FFV* of high pressure (5 MPa) is about three times larger than that of atmospheric pressure, while the *FFV* of high temperature (110°C) is 0.5 ∼ 1% larger than that of low temperature (50°C), thus the influence of pressure is greater than that of temperature figure 5.

### 3.4. Glass transition temperature

The *T*_g_ is a kind of common phenomenon of the polymer, and there is no perfect theory to exactly explain the experimental facts so far [27]. Under the *T*_g_, the molecular chain segment and the free volume are frozen and maintain at a constant value, and the sizes and distribution of free volume will remain basically fixed even with the increase of temperature. However, above the *T*_g_, the free volume begins to change from freezing into motion and the sizes of free volume gradually increase with the increase of temperature since the molecular chain segment has enough motion energy and begins to enter motion state. This may be due to the fact that the glass transition temperature is at a certain threshold temperature when the free volume reaches a critical value. Therefore, measuring the change of volume and the *FFV* with the temperature curve on the inflection point is a method to obtain *T*_g_.

**Figure 6.**
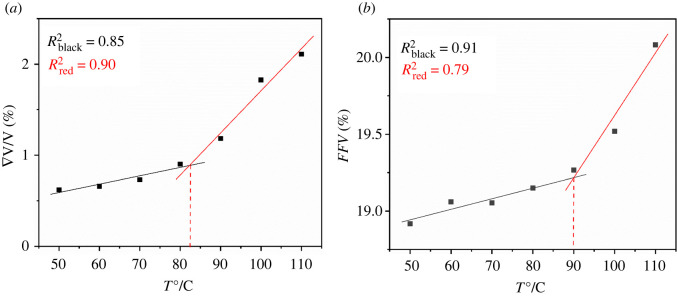
(*a*) The curves of rate of volume change with temperature for the pure PET (*b*) the curves of the FFV with temperature for the pure PET.

Figure 6*a* shows the relationship between temperature and rate of volume change for the pure PET without CO_2_. The preliminary rough estimation of the point of inflection is obtained by visual inspection of the simulated data points, which explains the determination of *T*_g_. Then, the two corresponding parts under the *T*_g_ and above the *T*_g_ were fitted into two straight lines by linear regression. The *T*_g_ of PET polymer, namely the intersection point of the two lines obtained by molecular simulation, was 83°C, which was close to the experimental *T*_g_ of 87°C [10]. Meanwhile, the curves of the *FFV* with temperature for the pure PET without CO_2_ are shown in figure 6*b*. Obviously, the *T*_g_ of the pure PET without CO_2_ was 90°C, which was a little slightly larger than the experimental *T*_g_ of 87°C.

**Figure 7.**
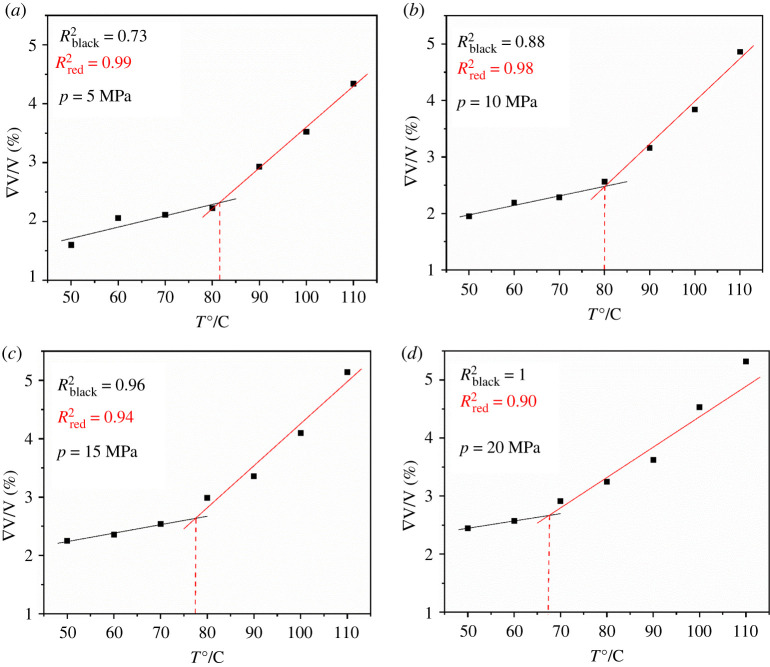
The curves of volume change with temperature for the PET at different pressure (*a*) *p* = 5 MPa, (*b*) *p* = 10 MPa, (*c*) *p* = 15 MPa, (*d*) *p* = 20 MPa.

**Figure 8.**
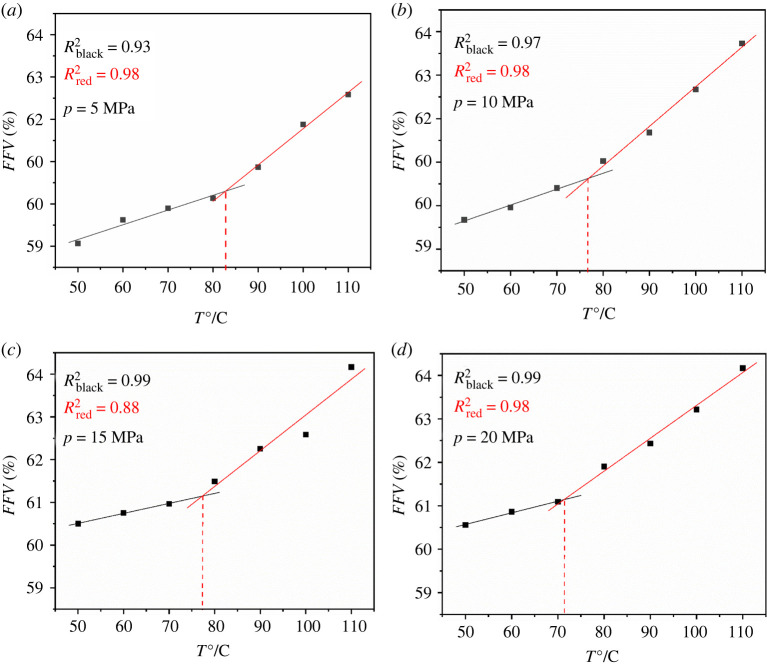
The curves of the *FFV* with temperature for the PET at different pressure (*a*) *p* = 5 MPa, (*b*) *p* = 10 MPa, (*c*) *p* = 15 MPa, (*d*) *p* = 20 MPa.

The kind of curves of volume change with temperature for the PET polymer at the pressure ranging from 5 to 20 MPa were obtained, as shown in figure 7. Similarly, for the *FFV* dependence of temperature for the swelled PET polymer, the same kinds of curves were obtained, as shown in figure 8. The glass transition temperature at different pressures shown in table 2 is in good agreement with literature [10]. The relative error temperature is less than 10°C. As the pressure increases, the error relative increases, even exceeding 10% (less than 15%). The crystallinity and thermal treatment of the polymer have an influence on the glass transition temperature. When simulating this process, the authors only established the amorphous region, but did not establish the crystallization region, resulting in a different *T*_g_ compared with the experimental value. In general, the higher the crystallinity is, the higher the *T*_g_. However, the simulation results are larger than the experimental results in the literature, and further experiments are needed to check the crystallinity of experimental materials by adsorption experiments and to determine the glass transition temperature.

**Table 2.** The glass transition temperature of PET at different pressures.

pressure/MPa	0.1		5		10		15		20	
*T*_g_ (°C)	by Δ*V*/*V*	by *FFV*	by Δ*V*/*V*	by *FFV*	by Δ*V*/*V*	by *FFV*	by Δ*V*/*V*	by *FFV*	by Δ*V*/*V*	by *FFV*
	82	90	81	83	80	77	77	76	67	71
literature [10] (°C)	87	82	75	68	62					
relative error (%)	−5.75%	3.45%	−1.22%	1.22%	6.67%	2.67%	13.2%	11.7%	8.06%	14.5%

### 3.5. Radial distribution functions

The radial distribution function (RDF) is an important physical quantity used to analyze and describe the whole equilibrium trajectory of the distribution of other molecules around a studied molecule. According to the position and intensity of the peak in the RDF, the accessibility and affinity of the adsorption site of a specific sorbate on the polymer can be determined. The typical atoms on the PET molecule chains are the single bond oxygen and double bond oxygen (labelled as O_1_ and O_2_, respectively).

**Figure 9.**
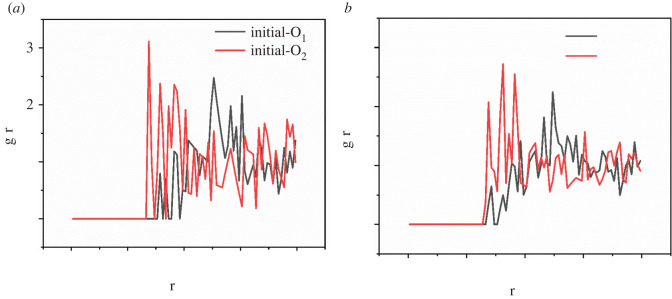
RDFs (g(r)) of CO_2_ around O_1_ and O_2_ atoms in PET at the beginning and ending of the sorption process. (*a*) The initial stages, (*b*) the final stages.

Figure 9 shows the RDFs of CO_2_ molecules calculated at 10 MPa and 100°C around the typical atoms (sorption sites) of the PET polymer. The results indicate that the obvious peak values of O_1_ sites and O_2_ sites were 5.05 and 2.75 at the beginning of the SRC (with 13 CO_2_ molecules/simulation cell), reflecting the O_2_ sites are preferentially adsorbed compared to O_1_ sites. Meanwhile, the obvious peak values of O_1_ sites and O_2_ sites were 4.95 and 3.25 at the ending of the SRC (with 35 CO_2_ molecules/simulation cell), revealing the O_2_ sites were preferentially adsorbed compared to O_1_ sites.

**Figure 10.**
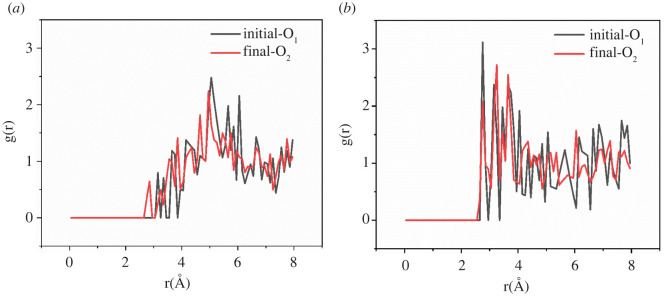
RDFs (g(r)) of CO_2_ around O_1_ and O_2_ atoms in PET at the beginning and ending of the sorption process. (*a*) RDF of O_1_ and (*b*) RDF of O_2_.

Figure 10 shows the change in the CO_2_–polymer interactions by comparing RDFs at the beginning and ending of the sorption process. The position of the first small and highest peak of CO_2_-O_1_ moves to the left, meanwhile, the position of the first peak of CO_2_–O_2_ is unchanged. However, the intensity of the first peak of CO_2_–O_2_ becomes smaller. The adsorption capacity of the O_1_ sites became stronger than before compared with the O_2_ sites, which indicated the reorientation of the main chain caused by the flexibility of the chain during the CO_2_ adsorption process. CO_2_ molecules occupying the sorption sites on the PET backbone increased the fluidity of the chain and then promoted plasticization [15].

**Figure 11.**
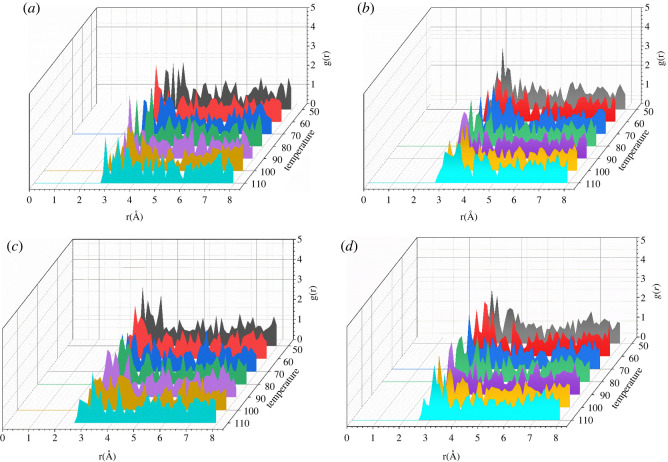
RDFs (g(r)) of CO_2_ around O_2_ atoms in PET at different pressure (*a*) *p* = 5 MPa, (*b*) *p* = 10 MPa, (*c*) *p* = 15 MPa, (*d*) *p* = 20 MPa.

**Figure 12.**
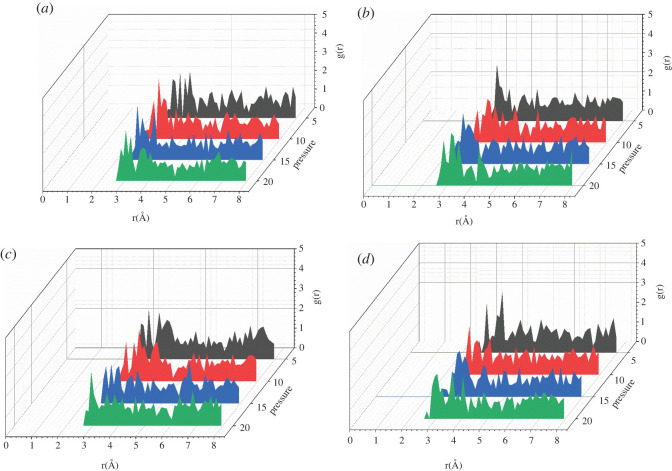
RDFs (g(r)) of CO_2_ around O_2_ atoms in PET at different temperature (*a*) *T* = 50°C, (*b*) *T* = 60°C, (*c*) *T* = 70°C, (*d*) *T* = 80°C.

**Figure 13.**
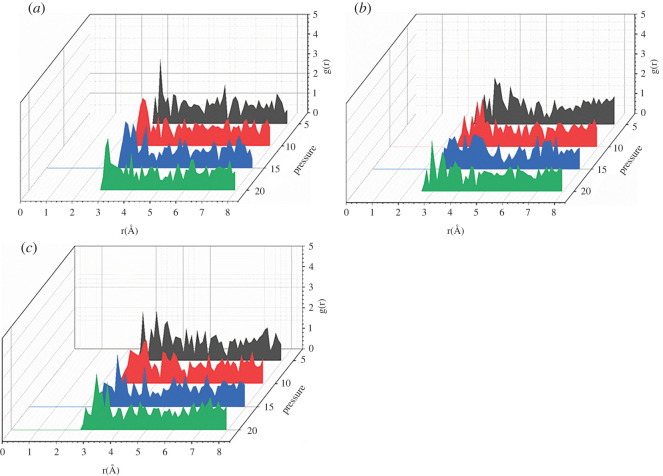
RDFs (g(r)) of CO_2_ around O_2_ atoms in PET at different temperature (*a*) *T* = 90°C, (*b*) *T* = 100°C, (*c*) *T* = 110°C.

Figure 11 shows the RDFs of CO_2_–O_2_ in the temperature range of 50 to 110°C. As the temperature increases, the position of the first peak of CO_2_–O_2_ moves to the right gradually and then moves to the left corresponding to the change trend reported in figure 3. Figures 12 and 13 show the RDFs of CO_2_–O_2_ in the pressure range of 5 to 20 MPa. The position of the first peak of CO_2_–O_2_ gradually moves to the left with the increase of pressure. The CO_2_–polymer interactions change from weak van der Waals force (3.1–5.0) to strong van der Waals force (2.6–3.1) and the adsorption capacity becomes stronger. With the higher pressure shown in figure 3, the adsorption concentration is correspondingly greater.

## 4. Conclusion

In the study, we predicted the adsorption concentration, swelling, *FFV*, and *T*_g_ of the PET polymer using the molecular dynamics simulation method. Firstly, the adsorption concentration reproduced through SRC was estimated in the same order of magnitude with the experimental data available in the reported literatures. Secondly, with the changes of temperature and pressure, the *FFV* induced by SC-CO_2_ in the PET polymer changes regularly, which was proportional to the capacity of SC-CO_2_ adsorption. Lastly, the *T*_g_ was further estimated almost identically to the known experimental values and shows a gradually decreased tendency with the increase of pressure. Thus, the changes of the PET backbone through the particular interactions with the sorption sites by the sorption of CO_2_ molecules promoted swelling and plasticization by increasing the *FFV*. Meanwhile, the plasticization of PET polymer studied by radial distribution functions showed that CO_2_ molecules occupying the sorption sites on the PET backbone increased the fluidity of the chain and promoted plasticization. In conclusion, in the absence of experimental data, MD simulation can be used to calculate the plasticization properties of PET polymers and other polymers.

## Data Availability

Our data are available from the Dryad Digital Repository: https://doi.org/10.5061/dryad.crjdfn35s [28].
